# Modular engineering of *E. coli* coculture for efficient production of resveratrol from glucose and arabinose mixture

**DOI:** 10.1016/j.synbio.2022.03.001

**Published:** 2022-03-15

**Authors:** Jia Li, Zetian Qiu, Guang-Rong Zhao

**Affiliations:** a Frontiers Science Center for Synthetic Biology and Key Laboratory of Systems Bioengineering (Ministry of Education), School of Chemical Engineering and Technology, Tianjin University, Yaguan Road 135, Jinnan District, Tianjin, 300350, China; b Georgia Tech Shenzhen Institute, Tianjin University, Dashi Road 1, Nanshan District, Shenzhen, 518055, China

**Keywords:** Resveratrol, Metabolic engineering, Synthetic biology, CRISPRi, Coculture, Resveratrol addiction

## Abstract

Resveratrol, a valuable plant-derived polyphenolic compound with various bioactivities, has been widely used in nutraceutical industries. Microbial production of resveratrol suffers from metabolic burden and low malonyl-CoA availability, which is a big challenge for synthetic biology. Herein, we took advantage of coculture engineering and divided the biosynthetic pathway of resveratrol into the upstream and downstream strains. By enhancing the supply of malonyl-CoA via CRISPRi system and fine-tuning the expression intensity of the synthetic pathway genes, we significantly improved the resveratrol productivity of the downstream strain. Furthermore, we developed a resveratrol addiction circuit that coupled the growth of the upstream strain and the resveratrol production of the downstream strain. The bidirectional interaction stabilized the coculture system and increased the production of resveratrol by 74%. Moreover, co-utilization of glucose and arabinose by the coculture system maintained the growth advantage of the downstream strain for production of resveratrol throughout the fermentation process. Under optimized conditions, the engineered *E. coli* coculture system produced 204.80 mg/L of resveratrol, 12.8-fold improvement over monoculture system. This study demonstrates the promising potential of coculture engineering for efficient production of natural products from biomass.

## 1 Introduction

Resveratrol (3,5,4′-trihydroxy-trans-stilbene) is a natural plant polyphenolic compound [1]. It is well known that resveratrol has antioxidant, anti-inflammatory, and anticancer activities [2] which is beneficial to human health of cardiovascular and neurological systems. Resveratrol also shows positive metabolic effects on anti-aging, anti-diabetes and elongates the survival of aging yeast [3], drosophila [4], and mice [5]. Recently, the novel target of resveratrol on caloric restriction was discovered, and it directly inhibited cAMP-dependent phosphodiesterase, triggering a cascade of energy-sensing metabolic events [6]. Because of its broad physiological and pharmacological properties, resveratrol become an attractive ingredient for the pharmaceutical, food supplement, nutraceutical, and cosmetic industries [7].

Production of resveratrol is mainly extracted from knotweed roots or grape seeds, which suffers the low content of resveratrol and the geographic dependence for good plant growing practice. Considering shortfall of resveratrol supply from natural sources, microbial production of resveratrol via synthetic biology and metabolic engineering has attracted worldwide interest [8]. The tyrosine ammonia lyase (TAL), p-coumarate: CoA ligase (4CL), and stilbene synthase (STS) consists of the heterologous biosynthetic pathway of resveratrol from the endogenous metabolite l-tyrosine and malonyl -CoA in host microbes. The *E. coli* monoculture was firstly explored, and the heterologous expression of the *TAL*, *4CL*, and *STS* genes led to biosynthesis of resveratrol using l-tyrosine or p-coumaric acid as precursor [[9], [10], [11], [12], [13]]. The de novo bioproduction of resveratrol from sugar is more attractive than from precursor. However, minor amounts of resveratrol were synthesized from glucose by engineered *E. coli* [14,15]. Recently, using glucose and malonate as carbon sources, the resveratrol titer was improved in engineered *E. coli* monoculture via increasing the precursor malonyl-CoA supply as well as optimizing expression of the *TAL* mRNA secondary structure [16]. Alternatively, the synthetic microbial coculture engineering, as the next generation strategy of synthetic biology, has been proven to be a more promising route to produce valuable biofuels [17,18], chemicals [19,20], and natural products [21,22]. For the case of resveratrol, the *E. coli* coculture produced 22.58 mg/L of resveratrol using glycerol as carbon source [23]. Further metabolic engineering of the pentose phosphate pathway for improving the biosynthesis of p-coumaric acid and the glycolytic pathway for enhancing biosynthesis of malonyl-CoA increased the production of resveratrol to 55.7 mg/L using glucose as carbon source [24]. Despite the more efforts made by researchers, resveratrol production in coculture is needed to improve.

**Fig. 1:**
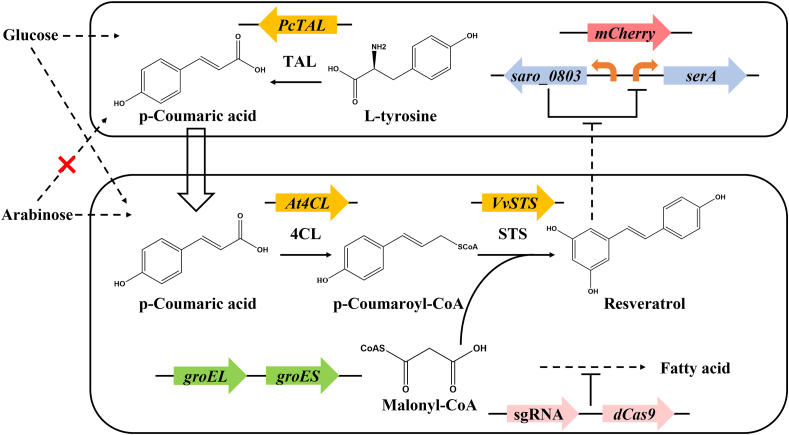
Schematic diagram of the *E. coli*-*E. coli* coculture to accommodate resveratrol biosynthetic pathway from glucose and arabinose mixture. TAL, tyrosine ammonia lyase; 4CL, p-coumarate: CoA ligase; STS, stilbene synthase; *saro_0803*, resveratrol biosensor gene; *serA*, phosphoglycerate dehydrogenase gene.

In this coculture engineering, we divided the de novo biosynthetic pathway of resveratrol into two *E. coli* strains (Fig. 1). The upstream strain harbored the *TAL* gene to synthesize p-coumaric acid from glucose, and the downstream strain harbored the *4CL* and *STS* genes for production of resveratrol from p-coumaric acid. To increase the intracellular availability of malonyl-CoA, we applied the RppA biosensor [25] for screening CRISPRi inhibitory targets. The upstream strain was constructed from arabinose-deficient and l-tyrosine-overproducer [26] by introducing the *PcTAL* gene [27]. To stabilize the subpopulation in coculture system, we designed and constructed a resveratrol addiction circuit in the upstream strain that coupled its growth with the resveratrol production of the downstream strain. After optimization of fermentation conditions, the coculture produced 204.80 mg/L of resveratrol with robust and compatible fashion using glucose and arabinose mixture.

## 2 Methods and materials

### 2.1 Chemicals and reagents

Resveratrol standard (≥98%, HPLC) was purchased from Solarbio (Beijing, China), and p-coumaric acid standard (≥98%, HPLC) was purchased from HEOWNS (Tianjin, China). Acetonitrile (HPLC grade) was purchased from Concord Tech (Tianjin, China). The ClonExpress II One-Step Cloning Kit was obtained from Biomed (Beijing, China) and DNA Polymerase of Phanta Super Fidelity and Rapid Taq Master Mix was obtained from Vazyme (Nanjing, China). Restriction enzymes and T4 DNA ligase were purchased from Thermo Scientific (USA). Purification of DNA, gel extraction, and plasmid preparation were conducted using kits from Tiangen Biotech Co., Ltd. (Beijing, China).

### 2.2 Construction of plasmids and strains

All strains and plasmids used in this study were listed in Table 1 and Table 2, respectively. All primers used in this study were listed in Supplementary Table S1. *E. coli* DH5α was used for propagation and cloning. For gene integration and deletion, *E. coli* genome were edited by CRISPR-Cas9 meditated method [28]. The T7 RNA polymerase gene was inserted into the locus between the *ybhB* and *ybhC* genes on *E. coli* MG1655 chromosome to obtain *E. coli* MG1655(DE3), followed by sequential deletion of the *endA* and *recA* genes to generate strains LJM1 and LJM2, respectively. By knocking out the *serA* gene on the chromosome of BAK11(DE3) which derived from *E. coli* BW25113, generating serine-deficient strain BAK21(DE3).

**Table 1:** Strains used in this study.

Strains	Description	Source
*E. coli* MG1655	*E. coli* K-12 F-λ-ilvG-rfb-50 rph-1	Invitrogen
MG1655(DE3)	*E. coli* MG1655 with T7 RNA polymerase gene in the chromosome	This study
LJM1	MG1655(DE3) Δ*endA*	This study
LJM2	LJM1 Δ*recA*	This study
BAK11	*E. coli* BW25113 Δ*ptsG*, Δ*tyrR*, Δ*pykA*, Δ*pykF*, Δ*pheA*, Δ*mao*-*paa* cluster::P_*lacUV5*_-*aroG*^*fbr*^-*tyrA*^*fbr*^-*aroE*, Δ*lacI*::P_T*rc*_-*ppsA*-*tktA*-*glk*	[33]
BAK11(DE3)	BAK11with P_*lacUV5*_-T7 RNA polymerase gene	[26]
BAK21(DE3)	BAK11(DE3) Δ*serA*	This study
LJM3	LJM2 harboring plasmid pLJ1	This study
LJM4	LJM2 harboring plasmid pLJ1 and pSGR	This study
LJM5	LJM2 harboring plasmid pLJ1 and pSGR25	This study
LJM6	LJM2 harboring plasmid pLJ1 and pSGR29	This study
LJM7	LJM2 harboring plasmid pLJ1 and pSGR19	This study
LJM8	LJM2 harboring plasmid pLJ1 and pSGR32	This study
LJM9	LJM2 harboring plasmid pLJ1 and pSGR9	This study
LJM10	LJM2 harboring plasmid pLJ1 and pSGR4	This study
LJM11	LJM2 harboring plasmid pLJ1 and pSGR15	This study
LJM12	LJM2 harboring plasmid pLJ1 and pSGR36	This study
LJM13	LJM2 harboring plasmid pLJ1 and pSGR37	This study
LJM14	LJM2 harboring plasmid pLJ1 and pSGR38	This study
LJM15	LJM5 harboring plasmid pLJ13	This study
LJM16	LJM5 harboring plasmid pLJ10	This study
LJM17	LJM5 harboring plasmid pLJ11	This study
LJM18	LJM5 harboring plasmid pLJ12	This study
LJM19	LJM5 harboring plasmid pLJ14	This study
LJM20	LJM2 harboring plasmid pLJ2, pLJ10 and pSGR35	This study
LJM21	LJM2 harboring plasmid pLJ3, pLJ10 and pSGR35	This study
LJM22	LJM2 harboring plasmid pLJ1, pLJ10 and pSGR34	This study
LJM23	LJM2 harboring plasmid pLJ2, pLJ10 and pSGR34	This study
LJM24	LJM2 harboring plasmid pLJ2, pLJ10 and pSGR25	This study
BCR1	BAK11(DE3) harboring plasmid pLJ5	This study
BCR2	BAK11(DE3) harboring plasmid pLJ6	This study
BCR3	BAK11(DE3) harboring plasmid pLJ7	This study
LJM28	BCR2 harboring plasmid pLJ2, pLJ10 and pSGR25	This study
LJM29	LJM2 harboring plasmid pLJ1 and pLJ9	This study
LJM30	LJM29 harboring plasmid pSGR25	This study
LJM31	LJM29 harboring plasmid pSGR29	This study
LJM32	LJM29 harboring plasmid pSGR9	This study
BCR4	BCR3 harboring plasmid pYG1 and pACYCDuet-1	This study
BCR5	BAK21(DE3) harboring plasmid pLJ8	This study
BCR6	BCR5 harboring plasmid pLJ7 and pYG1	This study
LJF1	LJM2 harboring plasmid pLJ4	This study
LJF2	LJF1 harboring plasmid pSGR	This study
LJF3	LJF1 harboring plasmid pSGR1	This study
LJF4	LJF1 harboring plasmid pSGR2	This study
LJF5	LJF1 harboring plasmid pSGR3	This study
LJF6	LJF1 harboring plasmid pSGR4	This study
LJF7	LJF1 harboring plasmid pSGR5	This study
LJF8	LJF1 harboring plasmid pSGR6	This study
LJF9	LJF1 harboring plasmid pSGR7	This study
LJF10	LJF1 harboring plasmid pSGR8	This study
LJF11	LJF1 harboring plasmid pSGR9	This study
LJF12	LJF1 harboring plasmid pSGR10	This study
LJF13	LJF1 harboring plasmid pSGR11	This study
LJF14	LJF1 harboring plasmid pSGR12	This study
LJF15	LJF1 harboring plasmid pSGR13	This study
LJF16	LJF1 harboring plasmid pSGR14	This study
LJF17	LJF1 harboring plasmid pSGR15	This study
LJF18	LJF1 harboring plasmid pSGR16	This study
LJF19	LJF1 harboring plasmid pSGR17	This study
LJF20	LJF1 harboring plasmid pSGR18	This study
LJF21	LJF1 harboring plasmid pSGR19	This study
LJF22	LJF1 harboring plasmid pSGR20	This study
LJF23	LJF1 harboring plasmid pSGR21	This study
LJF24	LJF1 harboring plasmid pSGR22	This study
LJF25	LJF1 harboring plasmid pSGR23	This study
LJF26	LJF1 harboring plasmid pSGR24	This study
LJF27	LJF1 harboring plasmid pSGR25	This study
LJF28	LJF1 harboring plasmid pSGR26	This study
LJF29	LJF1 harboring plasmid pSGR27	This study
LJF30	LJF1 harboring plasmid pSGR28	This study
LJF31	LJF1 harboring plasmid pSGR29	This study
LJF32	LJF1 harboring plasmid pSGR30	This study
LJF33	LJF1 harboring plasmid pSGR31	This study
LJF34	LJF1 harboring plasmid pSGR32	This study
LJF35	LJF1 harboring plasmid pSGR33	This study

**Table 2:** Plasmids used in this study.

Plasmids	Description	Source
pLJ1	pCDFDuet-1, P_T7_-*VvSTS*-P_T7_-*At4CL*, Str^r^	This study
pLJ2	pETDuet-1, P_T7_-*VvSTS*-P_T7_-*At4CL*, Amp^r^	This study
pLJ3	pRSFDuet-1, P_T7_-*VvSTS*-P_T7_-*At4CL*, Kan^r^	This study
pLJ4	pETDuet-1, P_T7_-*Sgr_RppA*, Amp^r^	This study
pLJ5	pACYCDuet-1, P_Trc_-*PcTAL*, Chl^r^	This study
pLJ6	pCDFDuet-1, P_Trc_-*PcTAL*, Str^r^	This study
pLJ7	pETDuet-1, P_Trc_-*PcTAL*, Amp^r^	This study
pLJ8	pACYCDuet-1, P_*saro_0803*_-*saro_0803*-P_*novl*_-*serA*, Chl^r^	This study
pLJ9	pETDuet-1, P_T7_-*CgaccBC*-P_T7_-*CgdtaR1*, Amp^r^	This study
pLJ10	pACYCDuet-1, P_T7_-*groEL-groES*, Chl^r^	This study
pLJ11	pACYCDuet-1, P_T7_-*dnaK-dnaJ*, Chl^r^	This study
pLJ12	pACYCDuet-1, P_T7_-*ibpA-ibpB*, Chl^r^	This study
pLJ13	pACYCDuet-1, P_T7_-*tig*, Chl^r^	This study
pLJ14	pACYCDuet-1, P_T7_-*clpB*, Chl^r^	This study
pSGR	pRSFDuet-1, P_T7_-*dCas9*, P_J23119_-sgRNA scaffold, Kan^r^	This study
pSGR1	pSGR derivate, P_J23119_-*gltA-1*-sgRNA scaffold	This study
pSGR2	pSGR derivate, P_J23119_-*gltA-2*-sgRNA scaffold	This study
pSGR3	pSGR derivate, P_J23119_-*gltA-3*-sgRNA scaffold	This study
pSGR4	pSGR derivate, P_J23119_-*sucC-1*-sgRNA scaffold	This study
pSGR5	pSGR derivate, P_J23119_-*sucC-2*-sgRNA scaffold	This study
pSGR6	pSGR derivate, P_J23119_-*sucC-3*-sgRNA scaffold	This study
pSGR7	pSGR derivate, P_J23119_-*fumC-1*-sgRNA scaffold	This study
pSGR8	pSGR derivate, P_J23119_-*fumC-2*-sgRNA scaffold	This study
pSGR9	pSGR derivate, P_J23119_-*fumC-3*-sgRNA scaffold	This study
pSGR10	pSGR derivate, P_J23119_-*mdh-1*-sgRNA scaffold	This study
pSGR11	pSGR derivate, P_J23119_-*mdh-2*-sgRNA scaffold	This study
pSGR12	pSGR derivate, P_J23119_-*mdh-3*-sgRNA scaffold	This study
pSGR13	pSGR derivate, P_J23119_-*aceB-1*-sgRNA scaffold	This study
pSGR14	pSGR derivate, P_J23119_-*aceB-2*-sgRNA scaffold	This study
pSGR15	pSGR derivate, P_J23119_-*aceB-3*-sgRNA scaffold	This study
pSGR16	pSGR derivate, P_J23119_-*adhE-1*-sgRNA scaffold	This study
pSGR17	pSGR derivate, P_J23119_-*adhE-2*-sgRNA scaffold	This study
pSGR18	pSGR derivate, P_J23119_-*adhE-3*-sgRNA scaffold	This study
pSGR19	pSGR derivate, P_J23119_-*fabD-1*-sgRNA scaffold	This study
pSGR20	pSGR derivate, P_J23119_-*fabD-2*-sgRNA scaffold	This study
pSGR21	pSGR derivate, P_J23119_-*fabD-3*-sgRNA scaffold	This study
pSGR22	pSGR derivate, P_J23119_-*fabH-1*-sgRNA scaffold	This study
pSGR23	pSGR derivate, P_J23119_-*fabH-2*-sgRNA scaffold	This study
pSGR24	pSGR derivate, P_J23119_-*fabH-3*-sgRNA scaffold	This study
pSGR25	pSGR derivate, P_J23119_-*fabB-1*-sgRNA scaffold	This study
pSGR26	pSGR derivate, P_J23119_-*fabB-2*-sgRNA scaffold	This study
pSGR27	pSGR derivate, P_J23119_-*fabB-3*-sgRNA scaffold	This study
pSGR28	pSGR derivate, P_J23119_-*fabF-1*-sgRNA scaffold	This study
pSGR29	pSGR derivate, P_J23119_-*fabF-2*-sgRNA scaffold	This study
pSGR30	pSGR derivate, P_J23119_-*fabF-3*-sgRNA scaffold	This study
pSGR31	pSGR derivate, P_J23119_-*pabA-1*-sgRNA scaffold	This study
pSGR32	pSGR derivate, P_J23119_-*pabA-2*-sgRNA scaffold	This study
pSGR33	pSGR derivate, P_J23119_-*pabA-3*-sgRNA scaffold	This study
pSGR34	pETDuet-1, P_T7_-*dCas9*, P_J23119_-*fabB-1*-sgRNA scaffold, Amp^r^	This study
pSGR35	pCDFDuet-1, P_T7_-*dCas9*, P_J23119_-*fabB-1*-sgRNA scaffold, Str^r^	This study
pSGR36	pSGR derivate, P_J23119_-*fabB-1*-sgRNA scaffold and P_J23119_-*fabF-2*-sgRNA scaffold	This study
pSGR37	pSGR derivate, P_J23119_-*fabB-1*-sgRNA scaffold and P_J23119_-*fumC-3*-sgRNA scaffold	This study
pSGR38	pSGR derivate, P_J23119_-*fabF-2*-sgRNA scaffold and P_J23119_-*fumC-3*-sgRNA scaffold	This study
pYG1	pRSFDuet-1, P_Trc_-*mCherry*, Kan^r^	This study

Genes *SgrppA* from *Streptomyces griseus* [25] (GenBank: BAE07216) and *Nasaro_0803* from *Novosphingobium aromaticivorans* DSM 12444 [29] (GenBank: ABD25248.1) were synthesized by GenScript (Nanjing, China). The resveratrol biosynthetic pathway genes were amplified from pCDF-T7-PcTAL-T7-At4CL and pET-T7-VvSTS [27], respectively. The *CgaccBC* and *CgdtsR1* genes were amplified from pRSF-acc [30]. Endogenous molecular chaperone genes (*groEL-groES*, *dnaK-dnaJ*, *ibpA/B*, *tig*, and *clpB*) were amplified from *E. coli* MG1655 chromosome by PCR. Plasmids for gene expression were constructed by PCR amplification and homologous recombination methods. For example, the *VvSTS* fragment was amplified using primers VvSTS-F and VvSTS-R, and an *At4CL*-carrying vector fragment was amplified from pCDF-T7-At4CL with primers VvSTS-ZT-F and VvSTS-ZT-R, then they were assembled to plasmid pLJ1 by 2 × Seamless Cloning Mix (Biomed, Beijing, China). Similarly, plasmids pLJ2 to pLJ14 were constructed, respectively.

To construct plasmid for expressing sgRNAs, the CRISPRi system backbone (with two *Bsa*I restriction sites) and the *dCas9* gene were assembled into the plasmid pRSFDuet-1 by homologous recombination method to obtain the starting vector pSGR. We used sgRNAcas9 (V3.0) [31] to design CRISPR sgRNA and evaluate potential off-target cleavage sites, and the design process conforms to the general sgRNA design workflow [32]. Specifically, the designed forward and reverse primers for each spacer were annealed to obtain a double-stranded inserted fragments, which could be cleaved by *Bsa*I and ligated into plasmid pSGR by T4 DNA ligase, resulting in the desired sgRNA expression plasmids pSGR1 to pSGR33. All inhibitory target sequences in this study were listed in Supplementary Table S2.

### 2.3 Media and fermentation conditions

Luria Bertani (LB) medium (5 g/L yeast extract, 10 g/L tryptone, and 10 g/L NaCl) was used for plasmid propagation and seed preparation. M9 medium (6 g/L Na_2_HPO_4_, 3 g/L KH_2_PO_4_, 0.5 g/L NaCl, 1 g/L NH_4_Cl, 1 mM MgSO_4_, and 0.1 mM CaCl_2_) was used for resveratrol production. For monoculture, M9 medium supplemented with 1 g/L yeast extract and 10 g/L glucose. For coculture, when the resveratrol addiction circuit was introduced, the fermentation medium was changed to M9 medium supplemented with 50 mg/L phenylalanine and 10 g/L glucose (or mixture of glucose and arabinose with desired amounts). As required, antibiotics were added to the culture medium at a final concentration of 30 μg/mL chloramphenicol, 50 μg/mL ampicillin, 30 μg/L kanamycin, and 30 μg/mL streptomycin.

For biosynthesis of resveratrol, all engineered strains were cultivated overnight at 37 °C and 220 rpm in 5 mL of LB medium, and then 500 μL of culture was transferred into 50 mL of LB medium in 250 mL shake flask and incubated at 37 °C again with shaking at 220 rpm for 6–8 h. Cells were collected by centrifugation and resuspended into 250 mL shake flask with 25 ml fermentation medium with an initial optical density at 600 nm (OD_600_) of 1.0 (unless otherwise specified), and 0.2 mM of isopropyl β-d-1-thiogalactopyranoside (IPTG) was added for induction of gene expression. The fermentation was carried out at 30 °C with shaking at 220 rpm to produce resveratrol. Broth sample was removed at 48 h (unless otherwise specified) for analysis of products.

### 2.4 Coculture system analysis

To measure the subpopulation in coculture system, the upstream strain was engineered to possess red fluorescence protein gene *mCherry*. The fermentation broth was periodically withdrawn and centrifuged, and the cell pellets were washed twice, adjusted the to 1.0 of OD_600_ with phosphate buffered saline (PBS) for measuring *mCherry* expression. Red fluorescence intensity was detected using 96-well black polystyrene plate by the microplate reader (SpectraMax M2, Molecular Devices, USA), with an excitation wavelength of 587 nm and emission wavelength of 610 nm. Then the fluorescence intensity of each sample was corrected by subtracting its background (strain without the *mCherry* gene). The upstream strain subpopulation in the coculture system was estimated with the fluorescence intensity of the coculture divided by that of the upstream strain monoculture.

### 2.5 Biomass and metabolite analysis

Cell growth of strains was measured by detecting the OD_600_ value using a T6 spectrometer (Purkinje General, Beijing, China), and the residual glucose was measured with an S-10 biosensor (Siemantec Technology, Shenzhen, China). When using mixed carbon sources for fermentation, glucose and arabinose were measured by a Morphling™ Sugar-H column (300 × 7.8 mm, 5 μm) and a RI detector with a mobile phase (5 mM H_2_SO_4_) at 0.6 mL/min, 65 °C.

For analysis of resveratrol and p-coumaric acid, 5 ml of the fermentation broth was removed and extracted with 3 ml ethyl acetate by vortex mixer for 2 h. The top organic layer was evaporated to dryness, and then dissolved in 0.5 mL of ethanol, and the solution was filtered through 0.2 μm syringe filter. Agilent 1200 HPLC system equipped with a C18 column (150 * 4.6 mm with a particle size of 5 μm) maintained at 35 °C and a PDA detector (Agilent). Resveratrol and p-coumaric acid were analyzed at 303 and 277 nm, respectively, using a solution (30% acetonitrile, 70% water, 0.1% trifluoroacetic acid) as mobile phase and a flow rate of 1.0 mL/min. The amount was calculated using a five-point calibration curve with the *R*^*2*^ coefficient higher than 0.99.

### 2.6 Malonyl-CoA biosensor characterization

For malonyl-CoA biosensor characterization, strain harboring reporting gene *RppA* was fermented for 16 h, the broth was centrifuged for 10 min at 4,000 rpm, and 150 μL of supernatant was transferred to a 96-well polystyrene plate to measure its optical density at 340 nm using a microplate reader (SpectraMax M2, Molecular Devices, USA). The cell pellets were resuspended in phosphate buffered saline (PBS) to measure OD at 600 nm. The biosensor signal was defined as OD_340_/OD_600_.

## 3 Results

### 3.1 Identification of target genes for enhancing malonyl-CoA using CRISPRi system

**Fig. 2:**
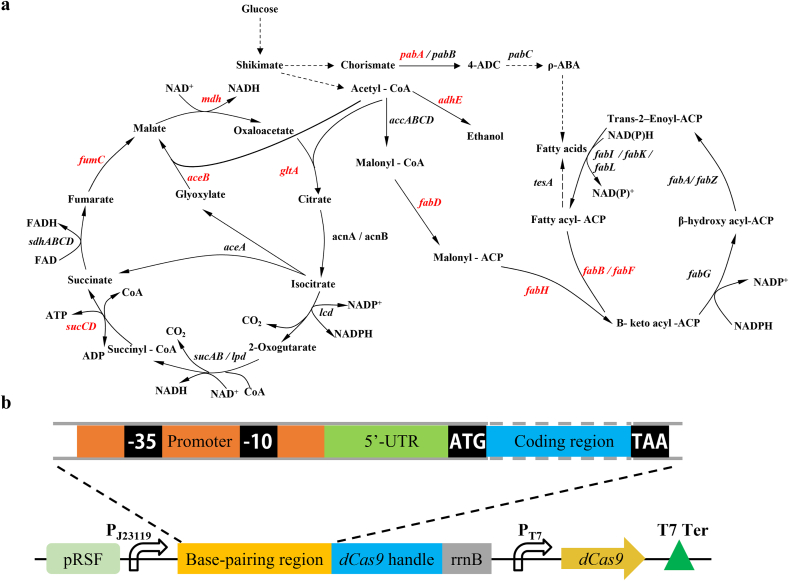
Gene targets for modulation by the CRISPRi system. (a) Schematic of the metabolic or regulatory pathways related to acetyl-CoA and malonyl-CoA metabolism in *E. coli*. Targeting genes are shown in red. (b) Schematic of the sgRNA blocking regions and *dCas9* expression in plasmid.

Three molecules of malonyl-CoA and one molecule of p-coumaroyl-CoA condensates to resveratrol. However, the limited supply of intracellular malonyl-CoA in *E. coli* significantly impedes the biosynthesis of resveratrol. The cellular malonyl-CoA derived from acetyl-CoA is used to synthesize fatty acids and phospholipids in microorganisms (Fig. 2a). In addition, acetyl-CoA is participated in tricarboxylic acid (TCA) cycle and formation of ethanol, resulting in the diversion of carbon flux away from malonyl-CoA (Fig. 2a). To enhance intracellular malonyl-CoA pool, we systematically adjusted the competition pathways of malonyl-CoA via CRISPR interference (CRISPRi) system. For increasing the availability of precursor acetyl-CoA, five genes (*gltA*, *sucC*, *fumC*, *mdh*, and *aceB*) in TCA circle and acetaldehyde dehydrogenase gene (*adhE*) were chosen as silencing targets. For preventing the diversion of malonyl-CoA to fatty acid biosynthesis, the *fabD*, *fabH*, *fabB*, and *fabF* genes were chosen as targets. In addition, knockdown of the *pabA* gene (encoding aminobenzoate synthetase) has been shown to be effective in increasing the pool of malonyl-CoA in *E. coli* for the increased titer of resveratrol [25]. In total, we chose 11 chromosomal genes that involves in the multiple pathways for modulation by CRISPRi (Fig. 2a). Targeting different sites in a gene allows the dCas9-sgRNA complex to exhibit different regulatory efficacy [34]. To obtain a better silence effect on each target gene, anti-sgRNAs sequences were designed by targeting the promoter region, 5′-untranslated region (5′-UTR), and/or 5′-terminal coding region (5′-TCR) (Supplementary Table S2). We constructed a library of 33 synthetic sgRNAs under controlled by J23119 promoter, and the *dCas9* gene was driven by T7 promoter (Fig. 2b).

**Fig. 3:**
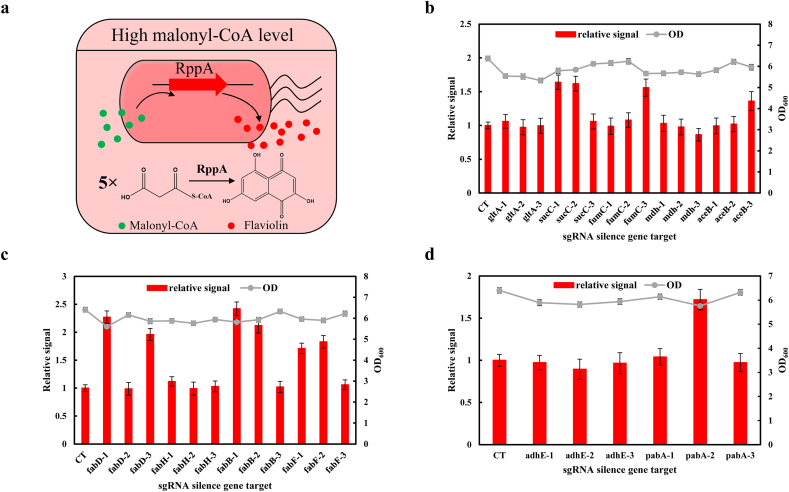
High-throughput screening of target genes with enhanced malonyl-CoA supply. (a) Schematic of the repurposed type III PKS RppA as a malonyl-CoA biosensor. (b) The inhibitory effects of TCA genes. (c) The inhibitory effects of fatty acid biosynthetic genes. (d) The inhibitory effects of genes in ethanol biosynthesis and chorismite pathway. CT: control strain LJF2. The three sgRNA sequences of the *gltA* and *sucC* genes targeted in promoter region, 5′-UTR, and 5′-TCR, respectively; three sgRNA sequences of the *fabD* and *fabF* genes targeted in 5′-TCRs, and sgRNA sequences of the rest seven genes (*fumC*, *mdh*, *aceB*, *fabH*, *fabB*, *adhE*, and *pabA*) targeted in one 5′-UTR and two 5′-TCRs.

To identify target genes with enhanced malonyl-CoA pool, we employed RppA (a type III polyketide synthase) which converts five molecules of malonyl-CoA into red-colored flaviolin (Fig. 3a) as malonyl-CoA biosensor [25] for high-throughput screening. Plasmid pLJ4 expressing the *RppA* gene was transformed into strain LJM2 to generate strain LJF1. Then we introduced plasmid harboring each of the 33 synthetic sgRNAs individually into strain LJF1 to implement gene perturbation. Plasmid pSGR without the target site complementary sequence was introduced into strain LJF1 to generate control strain LJF2, which did not modulate any chromosomal genes. As shown in Fig. 3b, for five genes of the TAC circle, the sgRNAs targeting *gltA* and *mdh* had no effects on the malonyl-CoA level, while the sgRNAs targeting *sucC-1*, *sucC-2*, *fumC-3*, and *aceB-3* increased 64%, 62%, 56%, 36% of malonyl-CoA level than the control, respectively. For silencing fatty acid pathway, the sgRNAs targeting *fabH* showed minor effect on malonyl-CoA level, while the sgRNAs targeting *fabD-1* (and *fabD-3*), *fabB-1* (and *fabB-2*), and *fabF-1* (and *fabF-2*) produced a dramatic increase in malonyl-CoA level (from 71% to 142%) (Fig. 3c). In addition, the sgRNA targeting *pabA-2* produced a 72% of increase in malonyl-CoA level, while sgRNAs targeting *adhE* had on effect (Fig. 3d).

Targeting different sites in the gene allows the dCas9-sgRNA complex to exhibit different regulatory effects. The inhibition of the *gltA*, *mdh*, *fabH*, and *adhE* genes by targeting three regions showed no effects on increasing the pool of malonyl-CoA, which were inconsistent with previous reports [16,35]. It might be due to targeting different sequences. Among the rest seven genes, the efficient inhibition was observed either in 5′-TCRs (*fumC-3, aceB-3, fabD-1, fabD-3, fabB-2, fabF-1, fabF-2,* and *pabA-2*), in 5′-UTRs (*sucC-2* and *fabB-1*), or in promoter region (*sucC-1*), instead of all regions of target gene. One possible reason is that the target sequence might determine the inhibitory effect. Nevertheless, all inhibition of aforementioned seven genes showed no significant difference in the final biomass. Hence, *fabB-1*, *fabD-1*, *fabF-2*, *pabA-2*, *sucC-1*, *fumC-3*, and *aceB-3* were chosen as the optimal inhibitory targets to produce resveratrol.

### 3.2 Enhancing resveratrol production by regulating malonyl-CoA metabolism

**Fig. 4:**
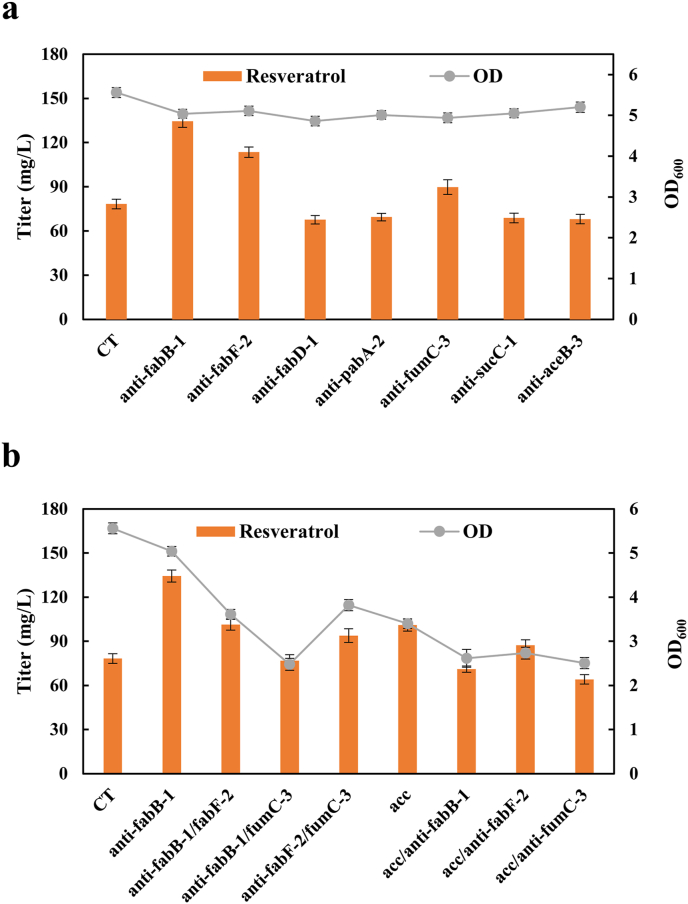
Effects of genetic perturbations on resveratrol production. (a) CRISPRi-based single target for resveratrol production. (b) Effect of combining targets on resveratrol production. 200 mg/L p-coumaric acid was supplemented in fermentation medium. CT: control strain LJM4.

We introduced plasmids harboring each of seven optimal sgRNAs individually into strain LJM3 to test changes in resveratrol production. Plasmid pSGR was introduced into the LJM3 strain to generate the control strain LJM4, which produced 78.32 mg/L resveratrol without modulation of any chromosomal genes. As shown in Fig. 4a, three inhibitory targets, *fabB-1*, *fabF-2*, and *fumC-3*, were validated to be beneficial for production of resveratrol. Specifically, the corresponding strains expressing anti-*fabB-1*, anti-*fabF-2*, and anti-*fumC-3* achieved resveratrol titers of 134.42, 113.49, and 89.75 mg/L, which were 72%, 45%, and 15% higher than that of the control strain LJM4, respectively, without obvious biomass change (lower than 12%). Furthermore, we explored the simultaneous interference of two genes by CRISPRi on resveratrol enrichment. However, combinatorial inhibition seemed unfavorable for resveratrol production, as all engineered strains exhibited lower titers of resveratrol and remarkably decrease in biomass (by over 24%), compared with targeting single gene (Fig. 4b). The possible reason is that the availability of malonyl-CoA was insufficient to maintain cell physiology, as cell growth was greatly inhibited upon strong inhibition of two targeting genes.

Acetyl-CoA carboxylase (ACC) catalyzes acetyl-CoA to malonyl-CoA. *E. coli* ACC consists of four subunits, and CgACC of *C. glutamicum* has two subunits (AccBC and DtsR1) which has been proven to be effective in increasing the pool of malonyl-CoA in *E. coli*, resulting in the increased titers of stilbenes and flavonoids [30]. As shown in Fig. 4b, when *CgaccBC*-*CgdtsR1* was expressed, resulting strain produced 101.06 mg/L of resveratrol, 29% increase compared with the strain LJM4, but an obvious decrease in biomass (by 39%) was observed. Then we combined the ACC complex with the CRISPRi system. Unfortunately, the titer of resveratrol did not further increase (Fig. 4b), probably because of inhibiting cell growth (decreased biomass by over 51%). Balanced allocation of malonyl-CoA between cell growth and heterologous molecule production is desirable to prevent the impairment of cell viability.

### 3.3 Combinatorial optimization of resveratrol production from p-coumaric acid in downstream strain

**Fig. 5:**
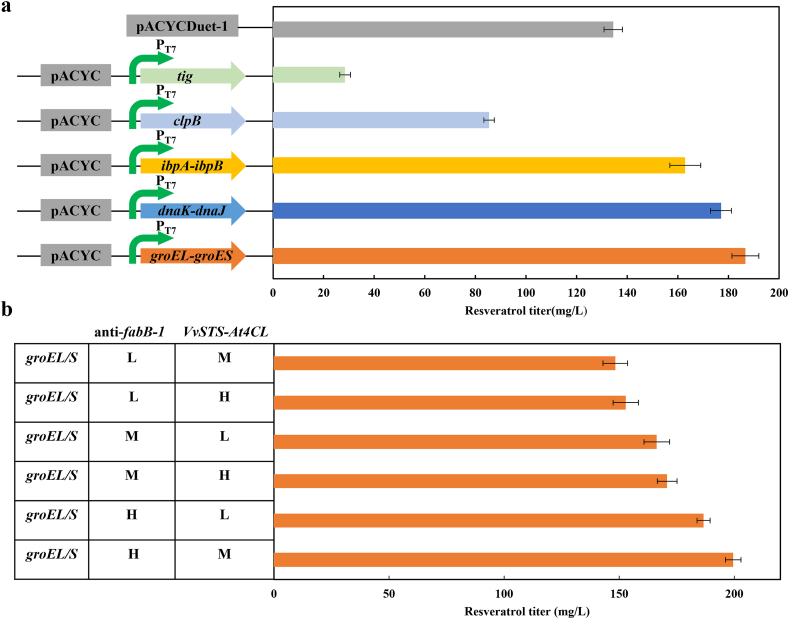
Combinatorial strategy to improve resveratrol titer from p-coumaric acid. (a) Screening chaperone overexpression for resveratrol production. (b) Optimizing resveratrol production by regulating the expression of resveratrol pathway genes (*VvSTS*-*At4CL*) and inhibitory target anti-*fabB-1* on various copy number plasmids. H: high-copy number (pRSFDuet-1). M: middle-copy-number (pETDuet-1). L: low-copy-number (pCDFDuet-1). 200 mg/L p-coumaric acid was added in fermentation medium.

We previously showed that overexpression of molecular chaperone *groEL-groES* gene was favored for production of resveratrol [27]. In order to screen best chaperone, the *tig*, *clpB*, *ibpA-ibpB*, and *dnaK-dnaJ* genes [36] were overexpressed in strain LJM5, respectively. As shown in Fig. 5a, the overexpression of the *tig* and *clpB* genes greatly decreased the titer of resveratrol, while the overexpression of *ibpA-ibpB* and *dnaK-dnaJ* enhanced resveratrol production of 162.92 mg/L and 177.08 mg/L, respectively, but they were less efficient than the overexpression of *groEL-groES,* which led to 186.66 mg/L of resveratrol, and was used in following optimization.

To further enhance the production efficiency of resveratrol from p-coumaric acid, it was necessary to regulate the expression intensities of the resveratrol pathway genes and inhibitory sgRNA. Anti-*fabB-1* and *VvSTS-At4CL* were expressed in low-, middle-, or high-copy number plasmids, and a total of six expression patterns were obtained. As shown in Fig. 5b, the higher copy numbers of anti-*fabB-1* led to higher production of resveratrol, when anti-*fabB-1* was expressed on low-copy-number plasmid, the production of resveratrol was lowest, which might be caused by the lack of intracellular malonyl-CoA level. While the copy number of *VvSTS-At4CL* did not exhibit a significant effect on the production of resveratrol, which further illustrated that malonyl-CoA posed a great role. Among all combinations, strain LJM24 showed the best resveratrol production capacity with 199.56 mg/L resveratrol.

### 3.4 Comparison of resveratrol production capacity in monoculture and coculture systems

**Fig. 6:**
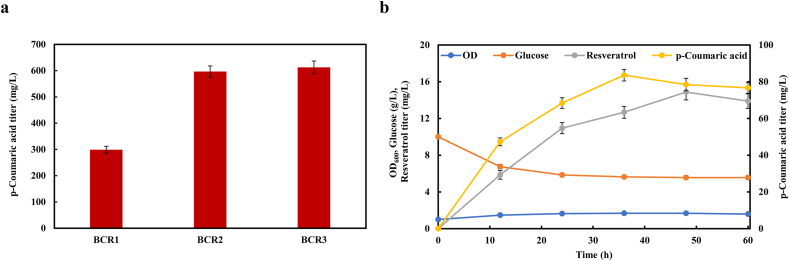
Metabolic engineering *E. coli* monoculture for production of resveratrol from glucose. (a) Production of p-coumaric acid by fine tuning of the *PcTAL* expression in *E. coli* BAK11(DE3). (b) Time profile of resveratrol fermentation in strain LJM28.

After resveratrol was efficiently produced from p-coumaric acid, we further extended the resveratrol biosynthesis from glucose. For producing p-coumaric acid from glucose, we used our previously reported l-tyrosine overproducing strain BAK11(DE3) [26] as chassis. For screening the expression pattern compatible with chassis, the *PcTAL* gene from *Phanerochaete chrysosporium* [27] under the control of the *trc* promoter was cloned into various copy number plasmids pACYCDuet-1, pCDFDuet-1, and pETDuet-1, and transformed into strain BAK11(DE3) to generate strains BCR1, BCR2, and BCR3, respectively. As shown in Fig. 6a, strain BCR1 produced 298.86 mg/L of p-coumaric acid, while strains BCR2 and BCR3 gave the almost the same amounts of p-coumaric acid (596.53 mg/L and 612.25 mg/L, respectively), over 200% of strain BCR1. It indicated that strain BCR2 would be sufficient for production of p-coumaric acid. For de novo biosynthesis of resveratrol from glucose, plasmids pLJ2, pLJ10 and pSGR25 were transformed into strain BCR2, resulting in strain LJM28. Resveratrol was successfully synthesized in strain LJM28 from glucose with the titer of 14.87 mg/L (Fig. 6b). However, cell growth was seriously retarded, and glucose consumption was very slow during the fermentation process (Fig. 6b). We speculated that the metabolic burden and conflicting metabolic goals were key factors leading to undesirable physiological changes, especially when multiple heterologous genes were overexpressed.

**Fig. 7:**
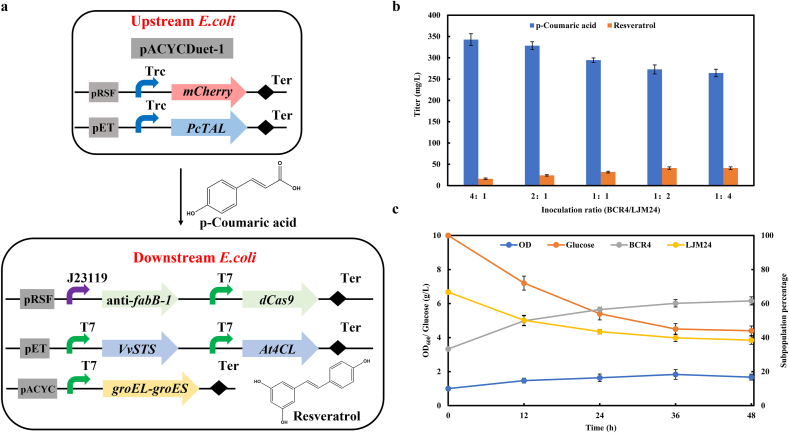
Resveratrol production in the *E. coli*−*E. coli* coculture system. (a) Schematic design of the two-strain coculture. (b) Optimization of resveratrol production by altering the inoculation ratio of strains BCR4 and LJM24. (c) Time profile of the overall cell density, sugar consumption, and subpopulation percentage in coculture of strains BCR4 and LJM24 with the initial BCR4/LJM24 ratio of 1/2.

To reduce the metabolic burden and possible mutual interference between the biosynthesis of p-coumaric acid and resveratrol, we divided the biosynthetic pathway of resveratrol into two separate strains. The aforementioned strain LJM24 harboring the downstream pathway was used for production of resveratrol from p-coumaric acid. For tracking population composition and balancing resistance between two strains, plasmids pYG1 (derived from pRSFDeut-1 backbone) expressing *mCherry* gene and pACYCDuet-1 were transformed into strain BCR3, resulting in the upstream strain BCR4 for producing p-coumaric acid from glucose (Fig. 7a). We first made efforts to balance the metabolic strength of two strains by adjusting the inoculation ratio of strains BCR4 to LJM24 (defined as the BCR4/LJM24 ratio) from 4:1 to 1:4 with an initial OD_600_ at 1.0. When the BCR4/LJM24 ratio was decreased, resveratrol production increased until the BCR4/LJM24 ratio at 1:2, where the highest production of resveratrol was achieved with a titer of 41.07 mg/L (Fig. 7b), nearly 3-fold of the monoculture. Then we analyzed the changes of total cell density, sugar consumption and population percentage in the coculture system with time under the optimal inoculation ratio at 1:2. As shown in Fig. 7c, the cell growth in the coculture system was poor and glucose consumption was very slow. Remarkably, the population of strain BCR4 increased from 33% to 60% after 48 h of fermentation (Fig. 7c), while strain LJM24 decreased from 67% to 40% (Fig. 7c), which indicates that two strains undergo competition and strain LJM24 exhibited a competitive disadvantage against strain BCR4.

### 3.5 Improving resveratrol production of coculture system by integrating resveratrol addiction circuit

**Fig. 8:**
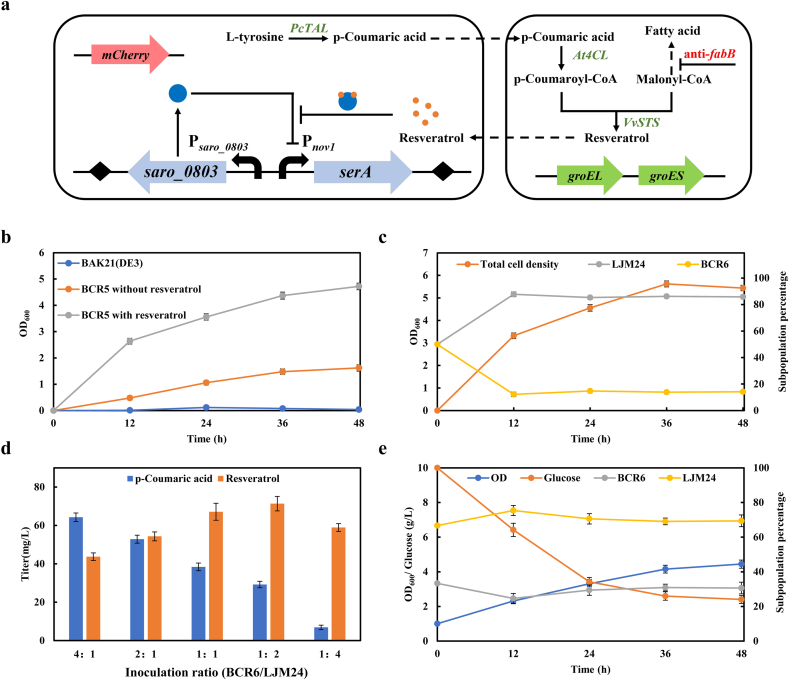
Improving resveratrol production by integrating resveratrol addiction circuit in the upstream strain. (a) Regulation pattern of P_*saro_0803*_-*saro_0803*-P_*novl*_-*serA* in the coculture. (b) Growth curve of strain BCR5 with or without resveratrol. BAK21(DE3) is a serine-deficient strain without resveratrol addiction circuit. (c) Time profile of the overall cell density, and subpopulation percentage of strains BCR6 and LJM24 at the initial ratio of 1/1 without induction of IPGT. (d) Optimization of resveratrol production by altering the inoculation ratios of strains BCR6 and LJM24. (e) Time profile of the overall cell density, sugar consumption, and subpopulation percentage of strains BCR6 and LJM24 with the initial ratio of 1/2 under IPTG induction.

In order to balance the subpopulations of two strains in coculture, we designed and constructed a resveratrol addiction circuit [29] in the upstream strain (Fig. 8a), wherein the growth of the upstream strain depended on resveratrol produced by the downstream strain. To validate the principle of resveratrol addiction circuit, we deleted the essential *serA* gene of strain BAK11(DE3) to generate the serine-auxotrophic strain BAK21(DE3), and introduced the resveratrol addiction circuit to construct strain BCR5. When cultivated in serine-deficient medium, strain BAK21(DE3) did not grow, and the expression of the *serA* gene under the control of resveratrol addiction circuit conferred the growth of serine-auxotrophic host BCR5 (Fig. 8b). Furthermore, adding 50 mg/L resveratrol to the medium enabled the cell growth of strain BCR5 to reach nearly 3-fold higher cell density (OD_600_) than without resveratrol supply (Fig. 8b), which indicated that there was a coupling relationship between resveratrol formation and cell growth. The upstream strain BCR6 was eventually generated by introducing *PcTAL* and *mCherry* into strain BCR5. In the BCR6-LJM24 coculture system, there might be a serine cross-feeding from strain LJM24 to strain BCR6, causing a faulted crosstalk between the sub-populations. Thus, the coculture without IPTG induction was carried out. A time course of the population dynamics showed that strain BCR6 could grow slowly, and its proportion was always at an absolute disadvantage (lower than 15%) (Fig. 8c). Moreover, serine and resveratrol were not detected in fermentation broth, which demonstrated that strain LJM24 without resveratrol biosynthesis had little effect on the growth of strain BCR6. Finally, the impact of the resveratrol addiction circuit on resveratrol production was evaluated in the BCR6-LJM24 coculture system at varying inoculation ratios. The result showed that 71.32 mg/L resveratrol was achieved at the optimal ratio of 1:2, 74% higher than without the resveratrol addiction circuit (Fig. 8d). During the fermentation process, the subpopulations of strains BCR6 and LJM24 were fluctuated at early growth stage and then maintained at ratio of 1:2 (Fig. 8e). More glucose was consumed, and the final cell optical density of the BCR6-LJM24 coculture was 1.4-fold higher than that of the BCR4-LJM24 without resveratrol addiction circuit (Fig. 7c).

### 3.6 Utilizing mixed carbon sources for efficient production of resveratrol

**Fig. 9:**
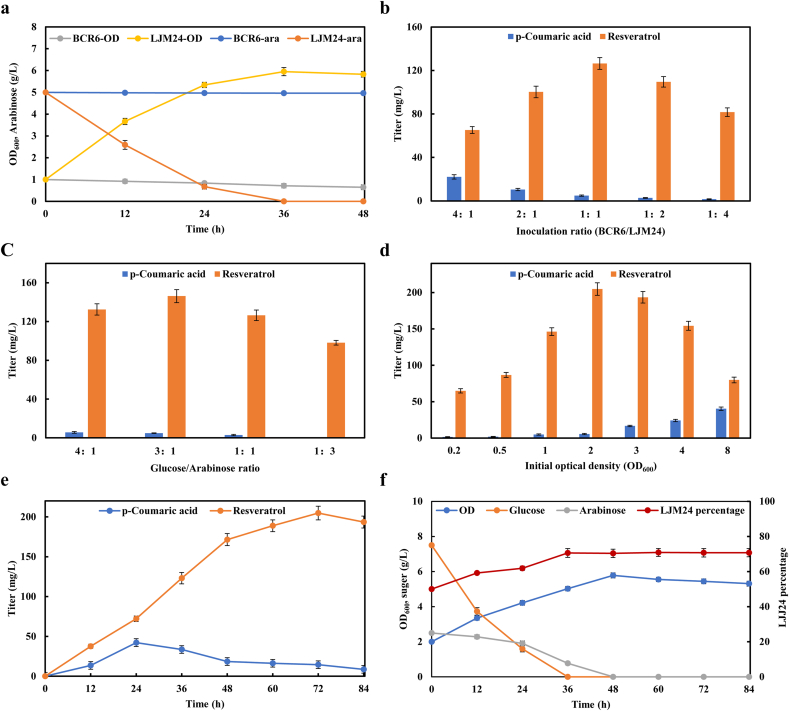
Improvement of resveratrol titer by co-utilization of glucose and arabinose. (a) Growth and sugar utilization of strains BCR6 and LJM24 using arabinose as sole carbon source. BCR6-ara and LJM24-ara represented the residual arabinose for strains BCR6 and LJM24, respectively. (b) Optimization of resveratrol production by altering the inoculation ratio of BCR6/LJM24 with the initial OD_600_ of 1.0 and the glucose/arabinose ratio of 1/1. (c) Optimization of resveratrol production by altering the ratio of glucose/arabinose with the initial OD_600_ of 1.0 and the initial BCR6/LJM24 ratio of 1/1. (d) Optimization of resveratrol production by altering the initial optical density with the glucose/arabinose ratio of 3/1 and the initial BCR6/LJM24 ratio of 1/1. (e) Time profile of p-coumaric acid and resveratrol production of batch fermentation with the initial OD_600_ of 2.0, the glucose/arabinose ratio of 3/1 and the initial BCR6/LJM24 ratio of 1/1. (f) Time profile of the overall cell density, sugar consumption, and LJM24% change with the initial OD_600_ of 2.0, the glucose/arabinose ratio of 3/1 and the initial BCR6/LJM24 ratio of 1/1.

Arabinose is the abundant sugar found in plant biomass and should therefore be potential attractive carbon source to produce valuable chemicals [37]. To enable strain LJM24 have a growth advantage in the coculture system, we tried to take arabinose with glucose as mixture carbon source to produce resveratrol. Especially, strain BCR6, derived from *E. coli* BW25113, is arabinose deficient as *araBAD* gene was deleted from the chromosome. We confirmed that strain BCR6 could not grow with arabinose as sole carbon source, while strain LJM24 could consume arabinose and smoothly grow (Fig. 9a).

We attempted to optimize the initial inoculation ratio of strains BCR6/LJM24 in the coculture at the glucose/arabinose ratio of 1/1 (5 g/L glucose and 5 g/L arabinose). As shown in Fig. 9b, when the inoculation ratio of BCR6/LJM24 was at 1:1, the highest production of resveratrol was achieved with a titer of 126.45 mg/L. Next, we attempted to balance the metabolic strength of two strains by varying the ratio of glucose to arabinose at the inoculation ratio of 1/1. As shown in Fig. 9c, the combinatorial profit of using the glucose and arabinose mixture was achieved at the ratio of 3/1 and the highest amount of resveratrol reached to 146.26 mg/L, which was 2.0-fold higher than glucose as the sole carbon source. Meanwhile the accumulation of intermediate p-coumaric acid was very low. To further improve the production of resveratrol, we optimized the initial inoculum size, and the maximum titer of 204.80 mg/L was achieved with an initial OD_600_ of 2.0 (Fig. 9d).

During the fermentation process of the coculture at optimized conditions, the titer of resveratrol increased gradually to the maximum at 72 h, and the amount of p-coumaric acid accumulated at low level after 24 h, representing a high efficiency of bioprocess (Fig. 9e). The dynamic change of subpopulation ratio of strains BCR6/LJM24 was correlated to the sugar consumption and resveratrol titer (Fig. 9f). During the first 24 h fermentation, both strains in coculture system preferentially used glucose, and strain LJM24 showed an obvious growth advantage compared with strain BCR6 (Fig. 9f). Meanwhile glucose was depleting, arabinose was available for maintaining strain LJM24, and the BCR6/LJM24 ratio was maintained at 1:2.4 (Fig. 9f). Our results indicated that an additional carbon source to maintain growth of strain LJM24 at the later stage of fermentation, making the maintain at a high ratio throughout the fermentation process.

## 4 Discussion

Malonyl-CoA is an important precursor for the biosynthesis of resveratrol. However, intracellular malonyl-CoA concentration usually maintains at very low level, which severely limits the production of resveratrol. Enhancing the supply of malonyl-CoA has been proved to increase the production of resveratrol in both monoculture and coculture system. The efforts to improve malonyl-CoA availability were mainly focused on ways to increase revenue and reduce expenditure of malonyl-CoA, including overexpression of ACC from *C. glutamicum* [9] to increase the conversion of acetyl-CoA into malonyl-CoA, introducing the recombinant malonate assimilation pathway (*matB* and *matC*) of malonyl-CoA from *Rhizobium trifolii* [12], fine-tuning the central metabolic pathways of malonyl-CoA metabolism [11,16,38]. Here, we took advantage of CRISPRi and RppA biosensor to identify potential targets that generally increased intracellular malonyl-CoA abundance. Among them, *fabB-1*, *fabF-2*, and *fumC-3* were validated to be beneficial for production of resveratrol, improving the titer of resveratrol without significantly inhibiting growth. However, it is noteworthy that increasing the supply of malonyl-CoA is not always beneficial for resveratrol production, as oversupply of malonyl-CoA could cause an undesired malonylation of the proteome, resulting in a further carbon burden in engineered *E. coli* [39]. Combining CRISPRi system with heterologous expression of ACC complex seemed unfavorable for resveratrol production, as all engineered strains exhibited remarkably decrease in biomass. It is agreed that the balanced distribution of malonyl-CoA between cell growth and resveratrol production is essential to increase resveratrol titer [11].

When individually optimized strains are physically mixed and cocultivated, their subpopulations in coculture system often change dynamically, and the coexistence may collapse due to the competitive growth and metabolic stress [20]. One strategy is to engineer competitive growth advantage that links cell growth with product formation. Metabolic addiction to maintain the growth adaptability is underexplored in metabolic engineering. Biosynthesis of mevalonic acid in addicted *E. coli* was controlled by fine-tuning of essential genes using mevalonic acid responsive transcription factor, which allowed the engineered strain to retain the high production capacity of mevalonate [40]. The similar strategy was used to stabilize the naringenin production phenotype of *Y. lipolytica* [41]. In this study, we demonstrated that the resveratrol addiction circuit could balance the subpopulations of two strains in coculture system by coupling resveratrol formation in one subpopulation to the cell growth of the other subpopulation. Implementation of resveratrol addiction circuit in a resveratrol-producing coculture resulted in a 74% increase of titer over varying inoculation ratios. Moreover, glucose consumption and the final biomass of the BCR6-LJM24 coculture system were remarkably improved.

When the strains co-cultivated on the sole carbon source, competition for growth would result in the incompatibility and instability of the consortium [42]. Coordinating cell subpopulations in coculture system through glucose-derepression is a promising strategy. Exploring glucose and xylose mixture effectively stabilizes *E. coli*-*E. coli* coculture for efficient production of muconic acid [19], salidroside [22],and rosmarinic acid [43]. In this study, we took glucose and arabinose mixture in coculture system, which enabled maintenance of the rational subpopulations of two strains and balanced cell growth and resveratrol production throughout the fermentation process, leading to increase of resveratrol titer.

In conclusion, redirecting malonyl-CoA to the resveratrol biosynthesis pathway via CRISPRi significantly increased the resveratrol synthesis ability from p-coumaric acid. Furthermore, resveratrol addiction circuit and glucose-arabinose mixture utilization improved coculture system compatibility and resveratrol yield. Under the optimal conditions in shake-flask fermentation, the coculture produced 204.80 mg/L of resveratrol with low accumulation of intermediate p-coumaric acid. Hence, the associated findings lay a foundation for future studies aiming at using these strategies to improve the production capacity of existing microbial coculture systems. Furthermore, the accomplishment of this study marks an important progress of modular coculture engineering for advancing microbial biosynthesis of valuable natural products.

## CRediT authorship contribution statement

**Jia Li:** Conceptualization, Investigation, Formal analysis, Visualization, Writing – original draft, Reviewing and Editing. **Zetian Qiu:** Investigation, Formal analysis, Writing – original draft. **Guang-Rong Zhao:** Conceptualization, Formal analysis, Visualization, Writing – review & editing, Supervision, Funding acquisition.

## Declaration of competing interest

The authors declare no competing financial interest.
