# Supplementary material for: Modular engineering of E. coli coculture for efficient production of resveratrol from glucose and arabinose mixture
Source: Synth Syst Biotechnol. 2022 Mar 15;7(2):718–29. doi: 10.1016/j.synbio.2022.03.001 (PMC8927788; doi:10.1016/j.synbio.2022.03.001)
Supplement: Multimedia component 1 [file mmc1.docx]

**Supporting Information**

Table S1. Primers used in this study

| Primers | Sequences |
| --- | --- |
| RppA-F | ATGGCGACCCTGTGCCGACC |
| RppA-R | TTAGCCGGACAGCGCAACGC |
| RppA-ZT-F | GCGTTGCGCTGTCCGGCTAAGGTACCCTCGAGTCTGGTAA |
| RppA-ZT-R | GGTCGGCACAGGGTCGCCATATGTATATCTCCTTCTTATA |
| VvSTS-F | ATGGCTTCAGTTGAGGAATT |
| VvSTS-R | TTAATTTGTAACCGTAGGAATGC |
| VvSTS-ZT-F | CATTCCTACGGTTACAAATTAACGAACAGAAAGTAATCGTAG |
| VvSTS-ZT-R | ATTCCTCAACTGAAGCCATGGTATATCTCCTTATTAAAGTT |
| At4CL-F | ATGGCGCCACAAGAACAAGC |
| At4CL-R | TTACAATCCATTTGCTAGTTTTGTCC |
| At4CL-ZT-F | AACTAGCAAATGGATTGTAAGGTACCCTCGAGTCTGGTAAAG |
| At4CL-ZT-R | GCTTGTTCTTGTGGCGCCATATGTATATCTCCTTCTTATACT |
| PcTAL-F | ATGCCTTCCCGCATCGACTA |
| PcTAL-R | CTACGCCTTGATAGACTTGA |
| PcTAL-ZT-F | TCAAGTCTATCAAGGCGTAGGGTACCCTCGAGTCTGGTAA |
| PcTAL-ZT-R | TAGTCGATGCGGGAAGGCATATGTATATCTCCTTCTTATACTTAAC |
| groEL/S-F | ATGAATATTCGTCCATTGCATGATCGC |
| groEL/S-R | TTACATCATGCCGCCCATGCCACCCAT |
| groEL/S-ZT-F | GCATGGGCGGCATGATGTAACCGCTGAGCAATAACTAGC |
| groEL/S-ZT-R | TGCAATGGACGAATATTCATATGTATATCTCCTTCTTATACTTAAC |
| dnak/J-F | ATGGGTAAAATAATTGGTATCGACC |
| dnak/J-R | TTAGCGGGTCAGGTCGTCAA |
| dnak/J-ZT-F | TTGACGACCTGACCCGCTAACCGCTGAGCAATAACTAGC |
| dnak/J-ZT-R | ATACCAATTATTTTACCCATATGTATATCTCCTTCTTATACTTAACT |
| ibpA/B-F | ATGCGTAACTTTGATTTATCCCCG |
| ibpA/B-R | TTAGCTATTTAACGCGGGAC |
| ibpA/B-ZT-F | GTCCCGCGTTAAATAGCTAACCGCTGAGCAATAACTAGC |
| ibpA/B-ZT-R | GATAAATCAAAGTTACGCATATGTATATCTCCTTCTTATACTTAAC |
| TF-F | ATGCAAGTTTCAGTTGAAACCAC |
| TF-R | TTACGCCTGCTGGTTCATCA |
| TF-ZT-F | TGATGAACCAGCAGGCGTAACCGCTGAGCAATAACTAGC |
| TF-ZT-R | GTTTCAACTGAAACTTGCATATGTATATCTCCTTCTTATACTTAACT |
| clpB-F | ATGCGTCTGGATCGTCTTAC |
| clpB-R | TTACTGGACGGCGACAATCC |
| clpB-ZT-F | GGATTGTCGCCGTCCAGTAACCGCTGAGCAATAACTAGC |
| clpB-ZT-R | GTAAGACGATCCAGACGCATATGTATATCTCCTTCTTATACTTAACT |
| dCas9-F | ATGGATAAGAAATACTCAATAGGC |
| dCas9-R | TCAGTCACCTCCTAGCTGAC |
| dCas9-ZT-F | GTCAGCTAGGAGGTGACTGACTCGAGTCTGGTAAAGAAAC |
| dCas9-ZT-R | GCCTATTGAGTATTTCTTATCCATATGTATATCTCCTTCTTATACTTAAC |
| accBC-F | GTGTCAGTCGAGACTAGGAA |
| accBC-R | TTACTTGATCTCGAGGAGAACAACG |
| accBC-ZT-F | CGTTGTTCTCCTCGAGATCAAGTAAGTCGAACAGAAAGTAATCG |
| accBC-ZT-R | CCTAGTCTCGACTGACACGGTATATCTCCTTCTTAAAGTTAAACAA |
| dtsR1-F | ATGACCATTTCCTCACCTTTGA |
| dtsR1-R | TTACAGTGGCATGTTGCCGT |
| dtsR1-ZT-F | ACGGCAACATGCCACTGTAAACCCTCGAGTCTGGTAAAGA |
| dtsR1-ZT-R | AAAGGTGAGGAAATGGTCATATGTATATCTCCTTCTTATACTTAAC |
| mCherry-F | ATGGTTTCAAAAGGTGAAGA |
| mCherry-R | TTATTTATATAATTCATCCATACCACC |
| mCherry-ZT-F | GGTGGTATGGATGAATTATATAAATAACCTCGAGTCTGGTAAAGAAA |
| mCherry-ZT-R | TCTTCACCTTTTGAAACCATATGTATATCTCCTTCTTATA |
| saro-F | TTATTCGTGAATGCGAGACAG |
| saro-R | AGTATATCTCCTCCTTGCAAT |
| saro-ZT-F | ATTGCAAGGAGGAGATATACTCCGCTGAGCAATAACTAGC |
| saro-ZT-R | CTGTCTCGCATTCACGAATAAAGGGAGAGCGTCGAGATCC |
| serA-F | ATGGCAAAGGTATCGCTGGA |
| serA-R | TTAGTACAGCAGACGGGCGC |
| serA-ZT-F | GCGCCCGTCTGCTGTACTAACCGCTGAGCAATAACTAGC |
| serA-ZT-R | TCCAGCGATACCTTTGCCATAGTATATCTCCTCCTTGCAAT |

Table S2. Inhibitory target sequence in this study

| Inhibitory target | Sequence (5′-3′)- | Inhibitory region |
| --- | --- | --- |
| gltA-1 | GCGAAGGCAAATTTAAGTTC | Promoter region |
| gltA-2 | TCTTACGCAATAAGGCGCTA | 5′UTR region |
| gltA-3 | AAAGCAAAACTCACCCTCAA | Coding region |
| sucC-1 | GACGTTTAACGTGTCTTATC | Promoter region |
| sucC-2 | CGGAAGCGATACGAAATATT | 5′UTR region |
| sucC-3 | TATGGCTTACCAGCACCGGT | Coding region |
| fumC-1 | AAATTAATCAGGTGAGGAGC | 5′UTR region |
| fumC-2 | ACGCAGCGAAAAAGATTCGA | Coding region |
| fumC-3 | ATGTCCCGGCAGATAAGCTG | Coding region |
| mdh-1 | GACTACACATTCTTGAGATG | 5′UTR region |
| mdh-2 | AGGATGAAAGTCGCAGTCCT | Coding region |
| mdh-3 | TTAAAAACCCAACTGCCTTC | Coding region |
| aceB-1 | CAACGATCCTTCGTTCACAG | 5′UTR region |
| aceB-2 | GAGCTGCACGATGACTGAAC | Coding region |
| aceB-3 | CCGATGAACTGGCTTTCACA | Coding region |
| adhE-1 | ACATTATCAGGAGAGCATTA | 5′UTR region |
| adhE-2 | TCGAATCCCACTCGCGAAAA | Coding region |
| adhE-3 | GCCGTTGCCGAATCCGGCAT | Coding region |
| fabD-1 | CAATTTGCATTTGTGTTCCC | Coding region |
| fabD-2 | GCATTTGTGTTCCCTGGACA | Coding region |
| fabD-3 | GGACAGGGTTCTCAAACCGT | Coding region |
| fabH-1 | AACTCGCAGTTTGCAAGTGA | 5′UTR region |
| fabH-2 | GCTATCTGCCCGAACAAGTG | Coding region |
| fabH-3 | AGTGCGGACAAACGCCGATT | Coding region |
| fabB-1 | ACTCTATGTGCGACTTACAG | 5′UTR region |
| fabB-2 | ATGAAACGTGCAGTGATTAC | Coding region |
| fabB-3 | CAGCATCGGTAATAACCAGC | Coding region |
| fabF-1 | AAGCGTCGTGTAGTTGTGAC | Coding region |
| fabF-2 | CTGGGCATGTTGTCTCCTGT | Coding region |
| fabF-3 | GCAATACCGTAGAGTCTACC | Coding region |
| pabA-1 | GGAATCTGAGTAAAATAGCG | 5′UTR region |
| pabA-2 | TGAACTGGGGGCGGATGTGC | Coding region |
| pabA-3 | CAACGATGCGTTGACGCTGG | Coding region |
